# Hsa_circ_0022383 promote non-small cell lung cancer tumorigenesis through regulating the miR-495-3p/KPNA2 axis

**DOI:** 10.1186/s12935-023-03068-5

**Published:** 2023-11-19

**Authors:** Xiaofang Xu, Binbin Song, Qiuliang Zhang, Weibo Qi, Yufen Xu

**Affiliations:** 1 Department of Oncology, The First Hospital of Jiaxing, Affiliated Hospital of Jiaxing University, No. 1882, Central South Road, Jiaxing, Zhejiang 314000 PR China; 2 Department of Nutriology, The First Hospital of Jiaxing, Affiliated Hospital of Jiaxing University, Jiaxing, Zhejiang 314000 PR China; 3 Department of Cardiothoracic Surgery, The First Hospital of Jiaxing, Affiliated Hospital of Jiaxing University, No. 1882, Central South Road, Jiaxing, Zhejiang 314000 PR China

**Keywords:** Non-small-cell lung cancer, Hsa_circ_0022383, MiR-495-3p, KPNA2

## Abstract

Hsa_circ_0022383 (circ_0022383) is a newly discovered circRNA. Its functions and relevant molecular mechanisms in tumorigenesis have not been reported. Here we aimed to explore how circ_0022383 regulates the tumorigenesis of non-small-cell lung cancer (NSCLC). We found thatcirc_0022383 expression was dramatically elevated in NSCLC tissues and cell lines. Upregulation of circ_0022383 was associated with poor prognosis in NSCLC patients. Silencing of circ_0022383 repressed cell proliferation and migration in vitro and inhibited oncogenesis and tumor metastasis in vivo. Moreover, our results discovered that circ_0022383 was mainly located in the cytoplasm of NSCLC cells. Mechanistically, circ_0022383 sponged miR-495-3p to modulate KPNA2 expression, thereby regulating NSCLC tumorigenesis and progression. In conclusion, our study demonstrates that circ_0022383 facilitates NSCLC tumorigenesis by regulating the miR-495-3p/KPNA2 axis, providing new insights into NSCLC development.

**Supplementary Information:**

The online version contains supplementary material available at 10.1186/s12935-023-03068-5.

## Introduction

Lung cancer (LC) is the most prevalent cancer in the respiratory system with high mortality and morbidity [1, 2]. Non-small-cell lung cancer (NSCLC) is a primary sub-type of LC, including lung squamous cell carcinoma, neuroendocrine cancer, large cell carcinoma, and lung adenocarcinoma, accounting for more than 80% of LC cases [3–5]. Because NSCLC has a high stake of relapse and metastasis [6], the clinical efficacy of current treatments is still unsatisfactory despite recent advances in NSCLC treatment [7]. Therefore, exploring the molecular mechanisms underlying NSCLC tumorigenesis and progression is a critical aspect of cancer biology.

Circular RNAs (circRNAs) are a novel type of non-coding RNAs (ncRNAs), characterized by a covalently closed loop that is back-spliced from an upstream 3’ site to a downstream 5 ' site of parental genes [8]. Due to the closed structure, circRNAs are more stable than linear RNAs and are resistant to digestion by RNA exonucleases [9]. Bioinformatics analysis and high-throughput sequencing have revealed that circRNAs are widely expressed across different cell types [10]. CircRNAs regulate diverse cellular processes, such as cell proliferation and migration [11–13]. Emerging evidence has revealed that circRNAs play a vital role in the tumorigenesis of malignant tumors, including NSCLC. For instance, circRNA_101237 facilities NSCLC tumorigenesis through regulating the miRNA-490-3p/MAPK1 signaling pathway [14]. CircRNA_103615 promotes the progression and cisplatin resistance of NSCLC by regulating ABCB1 expression [15]. CircP4HB contributes to NSCLC metastasis via sponging miR-133a-5p [16]. Circ_0022383 is a newly discovered circRNA derived from the exon 2 to 5 of *FADS2* located in chr11:61605249–61,615,756 with a genomic length of 10,507 bp. Yet, its functions and the relevant molecular mechanisms in NSCLC tumorigenesis remain largely unknown.

MicroRNAs (miRNAs) are small endogenous ncRNAs and repress gene expression through post-transcriptional regulation [17]. In tumorigenesis, miRNAs act as tumor suppressors or oncogenes [18–20].MiRNAs have been reported as important regulators of NSCLC progression [21–23]. For instance, miR-495-3p has been identified as a competing endogenous RNA (ceRNA) in LC tumorigenesis [24]. However, the role of miR-495-3p in NSCLC tumorigenesis remains elusive. Karyopherin subunit alpha 2 (KPNA2) belongs to the importin α family, transporting molecules including a canonical nuclear localization signal via forming an importin α/β/molecules heterotrimer [25, 26]. Considering its function in nucleocytoplasmic transport, KPNA2 is thought to play a crucial role in cell differentiation, proliferation, and migration [27]. Reportedly, KPNA2 facilitates NSCLC tumorigenesis [28–30]. However, whether KPNA2 functions as a miRNA target gene to regulate NSCLC tumorigenesis remains undetermined.

In this study, we aimed to unravel the role of circ_0022383 in NSCLC progression and to elucidate the relevant molecular mechanism.

## Materials and methods

### Clinical tissue samples

The primary NSCLC and adjacent normal tissues were obtained from 60 NSCLC patients by surgery at the Affiliated Hospital of Jiaxing University. The patients had not received any radiotherapy and chemotherapy before surgery. The tumor and health regions were confirmed by two professional pathologists. The dissected tumor and normal tissues were directly frozen in liquid nitrogen. Informed consent was obtained from all the enrolled patients. This study was approved by the Institutional Ethics Committee of the Affiliated Hospital of Jiaxing University and was performed following the Declaration of Helsinki [31].

### Cell culture and transfection

The four NSCLC cell lines (SPCA1, A549, CALU3, and H1299) and the human bronchial epithelial cell line (16HBE) were obtained from Sciencell (Sciencell, USA). All the cells were grown in the RPMI‑1640 medium (Hyclone, USA) containing 20% fetal bovine serum (FBS, Gibco, USA) at 37 ℃ with 5% CO_2_. The si-NC, si-circ_0022383, mimics control, miR-495-3p mimics, inhibitor control, and miR-495-3p inhibitor were bought from Genechem (Shanghai, China). The cells were transfected with indicated constructs using the Lipofectamine 3000 reagent (Invitrogen, USA) and then seeded into 6‑well plates.

### Cell cytosolic/nuclear fractions assay

The Cytoplasmic Nuclear RNA Purification kit (Norgen Biotek Corp, Canada) was used to isolate the nuclear and cytosolic fractions of NSCLC cells following the supplier’s instructions. Following RNA extraction from the nuclear and cytosolic fractions, the subcellular location of circ_0022383 in NSCLC cells was determined by qRT-PCR.

### RNase R treatment assay

The Cytoplasmic and Nuclear RNA Purification Kit (Thermo Fisher Scientific, USA) was employed to collect cytoplasmic and nuclear RNAs. The total RNA from tissues and cell lines was isolated by the TRIzol reagent (Invitrogen, USA). For the RNase R assay, the extracted total RNA of NSCLC cell lines was treated with RNase R (3 U/mg) for 15 min at 37 ℃, followed by qRT-PCR analysis.

### Quantitative real-time PCR assay

The total RNAs from NSCLC tissues and cell lines were collected using the Trizol reagent (Thermo Fisher Scientific, USA). A Reverse Transcription kit (Invitrogen, USA) was used for reverse transcription. PCR was performed on the Thermal Cycler Dice Real Time System (Takara, Japan) with the SYBR Green PCR master mix (Invitrogen, USA). Relative gene expression was measured through the 2^−ΔΔCt^ method. The primers for qRT-PCR are listed in Table 1. All experiments were carried out at least three times.

**Table 1:** Primers utilized for qPCR.

Gene	Forward 5′–3′	Reverse 5′–3′
Has_circ_0022383	5’-CGAGGGACA-GCAGTCAGAACA-3’	5’-GTGGCGGAGTCT-TCCCTTATT-3’
MiR-495-3p	5’- AGACAGAT-AGCCCGCAGAGG − 3’	5’-GATCTGCTGCCCTT-GTGCTGTC-3’
U6	5’-CGCT-TCCAGCACATATAC-3’	5’-CGCTTCGGCAGCAC-ATATAC-3’
KPNA2	5’- TGGTCTA-TGTCCGGTCCCATG-3’	5’- GCTGATTTGTGGGTG-TGGAA-3’
GAPDH	5’-GCGGGG-GAGCAAAGGGT-3’	5’- TGGGTGGCAGTGAT-GGCATGG-3’

### Western blot

Protein extraction from NSCLC cells was performed following a previously reported protocol [32]. Briefly, the protein was isolated by the RIPA lysis buffer (Invitrogen, USA) containing a protease inhibitor cocktail (PIC, Invitrogen, USA). The crude proteins were separated with a 10% SDS-PAGE gel and were then transferred to a PVDF membrane (Invitrogen, USA). Subsequently, the membrane was treated with 5% skim milk at room temperature for 2 h. After three washes in PBS, the membrane was treated with the primary antibody against KPNA2 (1:1000, Abcam, USA) and GAPDH (1:1000, CST, USA) at 4 ℃ overnight. Next, the membrane was treated with secondary antibodies (1:10000, Jackson, USA) at room temperature for 2 h. Finally, the ECL reagent (Amersham, UK) was applied to develop the blot. The band intensity was calculated using the ImageJ software (National Institutes of Health, USA).

### CCK‑8 assay

The CCK‑8 assay was performed to measure cell proliferation. The cells were first transfected with indicated constructs for 72 h and then were seeded into a 96‑well plate (1 × 10^4^ cells/well). Following incubation for 0 h, 24 h, 48 h, and 72 h, 10 uL of CCK‑8 reagent (Thermo Fisher Scientific, USA) was added to each well and further incubated at 37 ℃ for 2 h. The absorbance at 450 nm was calculated using a microplate reader (BioRad Laboratories, USA).

### Colony formation assay

For the colony formation assay, the cells were transfected with indicated constructs for 6 h and then were seeded into a 6-well plate (1 × 10^4^ cells/well). The cell colonies were first treated with 4% triformol (Invitrogen, USA), followed by staining with 0.1% crystal violet (Sigma, Germany). The colony number was calculated with a stereomicroscope (Leica, Germany).

### Transwell assay

After transfection with indicated constructs for 72 h, the cells were suspended (1 × 10^4^ cells/well) and applied to the top chamber. Then, the RPMI‑1640 medium containing 20% FBS was added to the low chamber. After incubation for 24 h, the cells were treated with 4% triformol (Invitrogen, USA), followed by staining with 1.5% crystal violet (Sigma, Germany) at 37 ℃. The number of migrated cells was determined under a stereomicroscope (Leica, Germany).

### Luciferase reporter assay

Luciferase reporter vectors, circ_0022383 wild-type (circ_0022383-WT), circ_0022383 mutant (circ_0022383-Mut), KPNA2 wild-type (KPNA2-WT), and KPNA2 mutant (KPNA2-Mut) were cloned into the pGL3-basic vector (Genechem, China). The vectors were co-transfected with mimics control or miR-495-3p mimics using the Lipofectamine 3000 reagent (Invitrogen, USA). After cell transfection with indicated constructs for 72 h, the transfected cells were harvested. Relative luciferase activities (Promega, USA) were calculated using the Luciferase Reporter Assay System (Ashland, USA). Each experiment was repeated at least three times.

### RNA pull-down assay

The relationship between circ_0022383 and miR-495-3p was verified using the RNA pull-down assay. For circ_0022383 pulled down miR-495-3p, the cells were transfected with circ_0022383 probe or NC probe. After transfection with indicated constructs for 48 h, the cells were harvested and treated with magnetic beads (Invitrogen, USA). After three washes in PBS, the miR-495-3p expression level was calculated by qRT-PCR.

### RNA immunoprecipitation (RIP) assay

The relationship between circ_0022383, miR-495-3p, and KPNA2 in NSCLC cells was investigated using the RNA immunoprecipitation assay. In brief, the cells were treated with the RIP lysis buffer. The control IgG antibody (1:1000; CST; USA) and anti‑Ago2 antibody (1:1000; CST; USA) were conjugated to magnetic beads, followed by incubation with cell extracts at 4 ℃ overnight. Subsequently, the magnetic beads were collected and treated with proteinase K. The enrichment of circ_0022383, miR-495-3p, and KPNA2 in immunoprecipitated RNA was calculated using qRT-PCR.

### Xenograft tumorigenesis

The nude mice were obtained from the Model Animal Research Center of Nanjing University (Nanjing, China). The animal protocol was approved by the Ethics Review Committee of the Affiliated Hospital of Jiaxing University (approval number: [KY-E-2022-06-27]). The NSCLC cell lines (A549) (1 × 10^6^/ mice) were transfected with si-NC or si-circ_0022383 plasmids suspended in PBS and were hypodermically injected into the mice. The tumor volume was calculated weekly. After 35 days, the mice were euthanatized by mask inhalation of 2% isoflurane and were sacrificed by cervical dislocation. Finally, the tumor tissue was weighed.

### Statistical analysis

All data were analyzed using the Prism 7.0 software and were presented as mean ± SD. Data analysis between the two groups was calculated using the Student’s *t* test. The significance among multiple groups was compared by ANOVA. The survival curve was established using the Kaplan–Meier plot. *P* < 0.05 was considered statistically different.

## Results

### Hsa_circ_0022383 expression in NSCLC tissues is upregulated

**Fig. 1:**
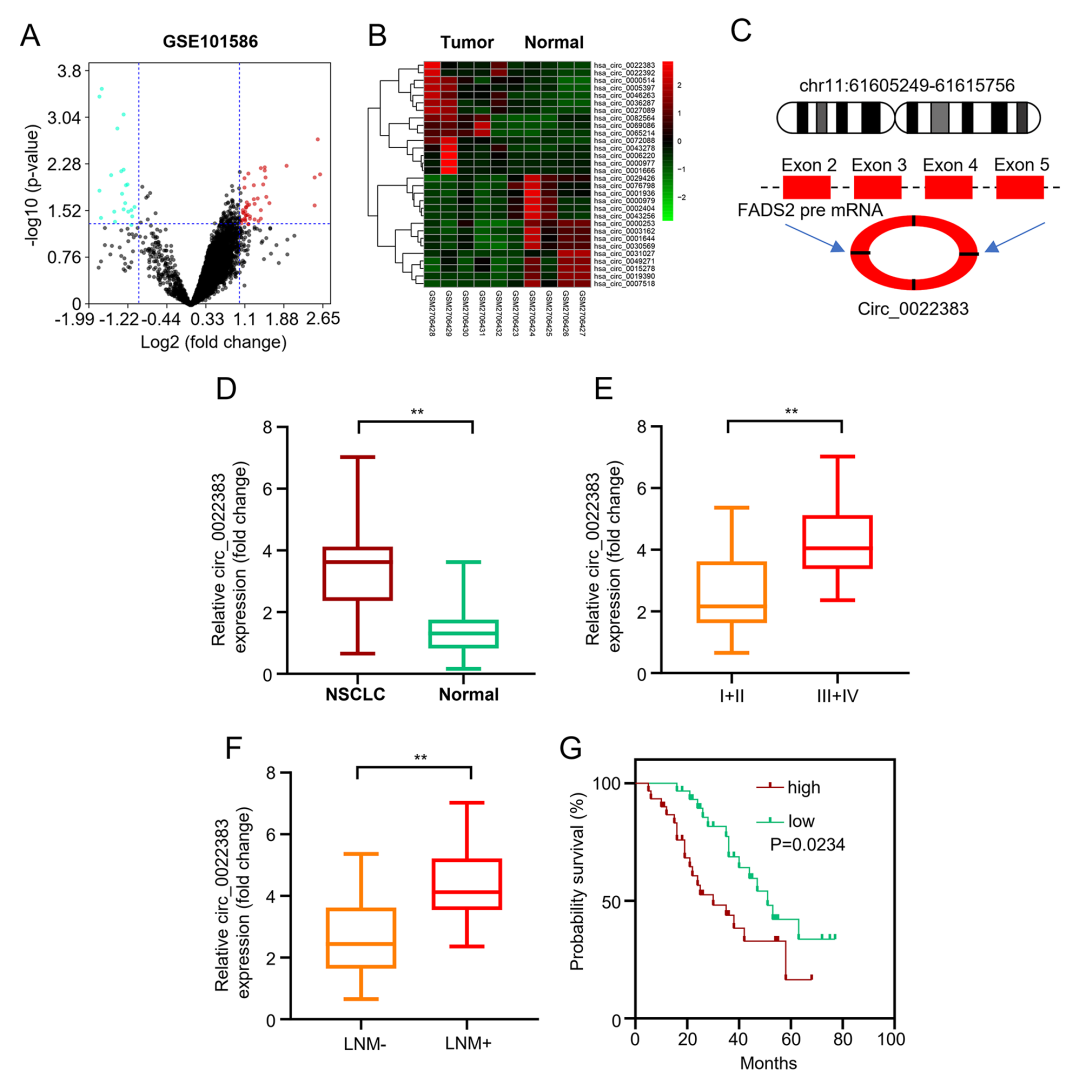
Hsa_circ_0022383 expression in NSCLC tissues is dramatically upregulated. **A**. Volcano plot of differentially expressed circRNAs in 5 pairs of primary NSCLC tissues and adjacent nontumor tissues in GEO database. **B**. Clustered heatmap presenting differentially expressed circRNAs in 5 pairs of primary NSCLC tissues as well as adjacent nontumor tissues in GEO database. **C**. Schematic illustration of circ_0022383 formation from *FADS2* through back splicing. **D**. Circ_0022383 expression in 60 pairs of primary NSCLC tumor tissues and adjacent nontumor tissues was calculated by qRT-PCR. **E**. Circ_0022383 expression in NSCLC tissues at different TNM stages was determined using qRT-PCR. **F**. Circ_0022383 expression in NSCLC tissues with or without lymph node metastasis was determined through qRT-PCR. **G**. Kaplan–Meier survival analysis of NSCLC patients with high or low circ_0022383 expression. All experiments were carried out in triplicate. “**” represents *P* < 0.01

We first used the GEO database to analyze circRNA expressions in NSCLC tissues. In total, we recovered 68 differentially expressed circRNAs (47 up-regulated and 21 down-regulated) in NSCLC tissues (*n* = 5) relative to adjacent tissues (*n* = 5), and circ_0022383 expression was markedly elevated in NSCLC tissues (Fig. 1A&B). Using a bioinformatics method, we found that circ_0022383 was formed from the exon 2 to 5 of *FADS2* (Fig. 1C). Using qRT-PCR, we detected circ_0022383 expression in 60 pairs of primary NSCL and adjacent tissues. Circ_0022383 expression was significantly higher in NSCLC than in adjacent tissues (Fig. 1D). Moreover, we found that the upregulation of circ_0022383 was closely associated with the tumor stage III and IV (Fig. 1E) and lymph node metastasis (Fig. 1F). Based on the median value of circ_0022383 expression, the 60 patients were divided into two groups, i.e., circ_0022383 low and high expression. The Kaplan-Meier analysis exhibited that circ_0022383 expression was positively correlated with poor prognosis in NSCLC patients (Fig. 1G). Together, these results suggest that circ_0022383 is highly expressed in NSCLC tissues and is involved in tumorigenesis.

### Formation, expression, structural characteristic of hsa_circ_0022383 in NSCLC cell lines

**Fig. 2:**
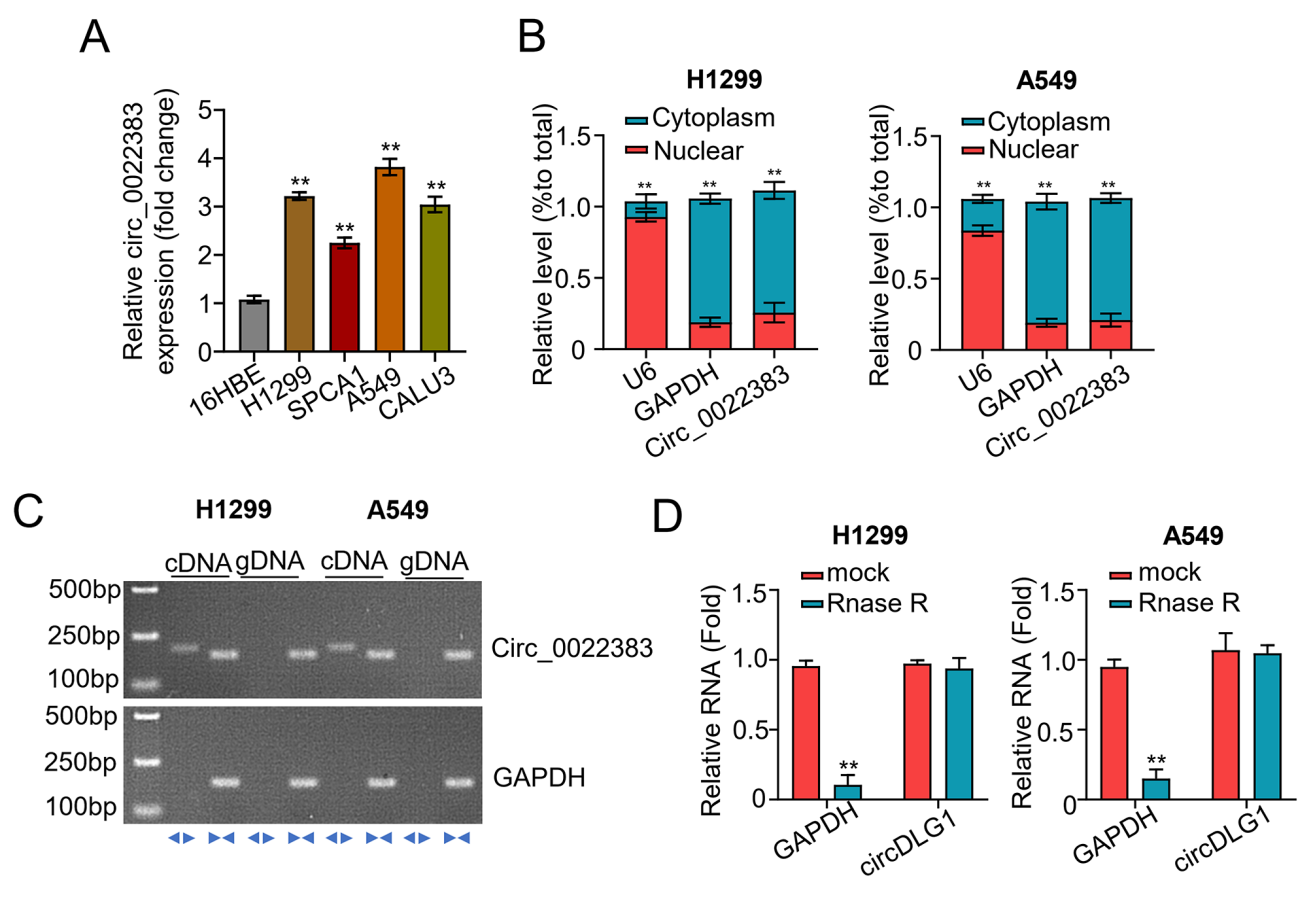
Characteristic of hsa_circ_0022383 in NSCLC cell lines. **A**. Circ_0022383 expression in four NSCLC cell lines (SPCA1, A549, CALU3 and H1299) and human bronchial epithelial cells (16HBE) was calculated by qRT-PCR. **B**. Circ_0022383 subcellular location in NSCLC cell lines (H1299 and A549) was detected through the cell cytosolic/nuclear fractions assay. **C**. Circ_0022383 expression was confirmed in NSCLC cell lines (H1299 and A549). **D**. Expression of circ_0022383 and FADS2 in NSCLC cell lines (H1299 and A549) without or with RNase R treatment was determined by qRT-PCR. All experiments were carried out in triplicate. “**” represents *P* < 0.01

We further detected circ_0022383 expression levels in four NSCLC cell lines (SPCA1, A549, CALU3, and H1299) and the human bronchial epithelial cell line (16HBE) using qRT-PCR. Circ_0022383 expression was significantly elevated in the four NSCLC cell lines with the highest level in A549 and H1299 cells (Fig. 2A). Further, we determined the subcellular localization of circ0022383 in A549 and H1299 cell lines using the cell cytosolic/nuclear fractions assay. We observed that circ_0022383 was mainly located in the cytoplasm (Fig. 2B). Next, we attempted to amplify circ_0022383 and *FADS2* from cDNA and gDNA using divergent and convergent primers. The PCR results revealed that circ_0022383 were detected in both cDNA and gDNA, while the *FADS2* only existed in the cDNA (Fig. 2C). In addition, using the RNase R assay, we found that the linear *FADS2* mRNA, not circ_0022383, was readily digested by RNase R (Fig. 2D), thus confirming the circular nature of circ_0022383.

### Circ_0022383 promotes NSCLC tumorigenesis

**Fig. 3:**
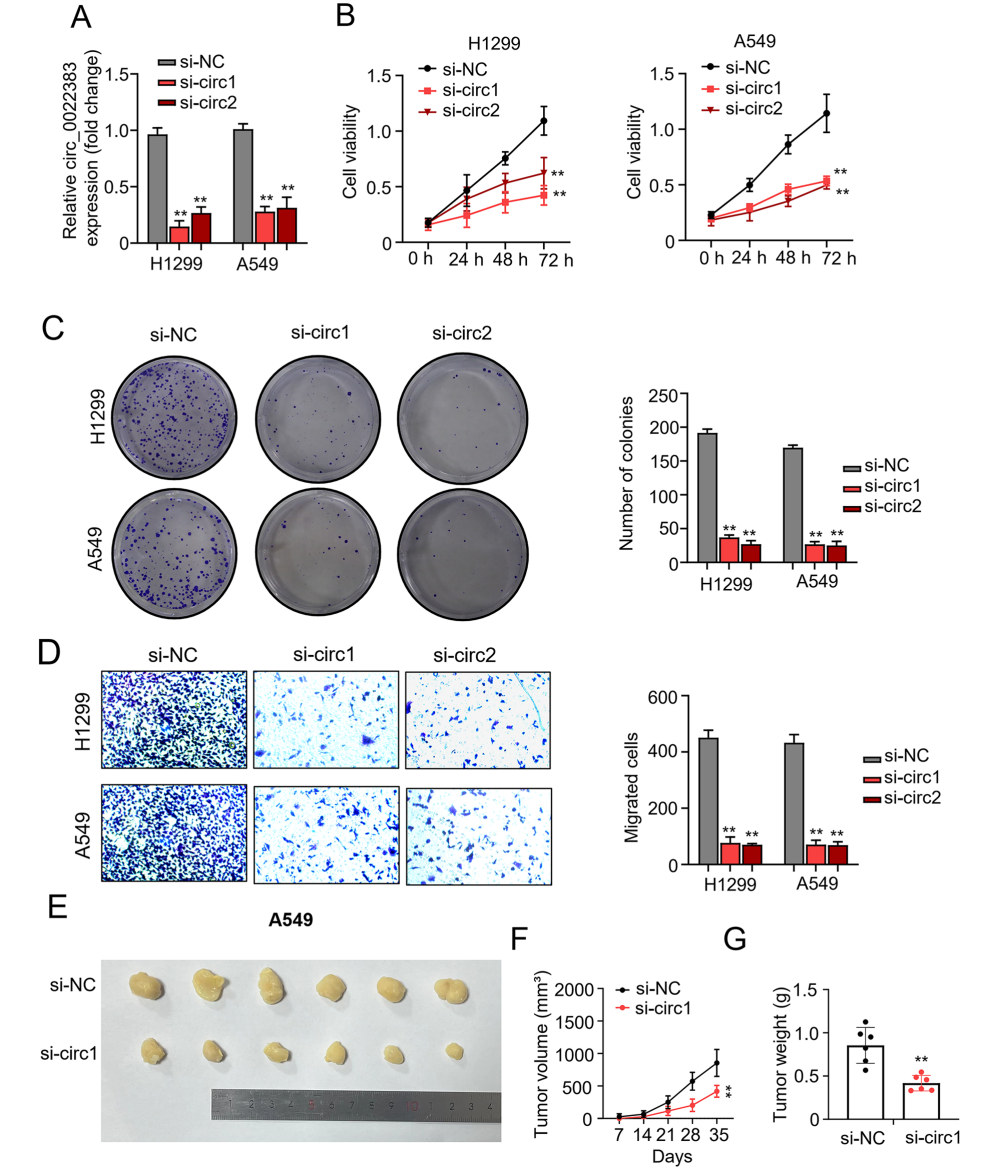
Hsa_circ_0022383 promotes NSCLC tumorigenesis. **A**. Circ_0022383 expression in NSCLC cell lines (H1299 and A549) transfected with si-NC or si-circ_0022383 was calculated by qRT-PCR. **B**. Cell proliferation in (A) was determined by the CCK-8 assay. **C**. Cell colony number in (A) was calculated by the colony formation assay. **D**. Cell migration capability in (A) was determined using the transwell assay. **E**. Tumor formation and growth in nude mice injected with NSCLC A549 cells transfected with si-NC or si-circ_0022383). **F**. Tumor volumes of nude mice injected with NSCLC A549 cells transfected with si-NC or si-circ_0022383. **G**. Tumor weight of nude mice injected with NSCLC A549 cells transfected with si-NC or si-circ_0022383. All experiments were carried out in triplicate. “**” represents *P* < 0.01

To further investigate the function of circ_0022383 in NSCLC, we silenced circ_0022383 expression in A549 and H1299 cells (Fig. 3A) and used the CCK-8 and colony formation assay to determine the cell proliferation ability. Silencing of circ_0022383 expression repressed cell proliferation and reduced cell colony numbers (Fig. 3B&C). In addition, we performed the transwell assay to determine cell metastasis abilities. Our results discovered that downregulation of circ_0022383 inhibited the metastasis potential of NSCLC cells (Fig. 3D). Moreover, we established the nude mice model by hypodermic injection of A549 cells transfected with si-NC or si-circ_0022383. The tumors with circ_0022383 silencing exhibited lower growth rates and lighter tumor weights compared to the control tumors (Fig. 3E-G). Collectively, these results indicate that circ_0022383 might function as an oncogene in NSCLC tumorigenesis.

### Circ_0022383 regulates KPNA2 expression by sponging mir-495-3p in NSCLC cells

**Fig. 4:**
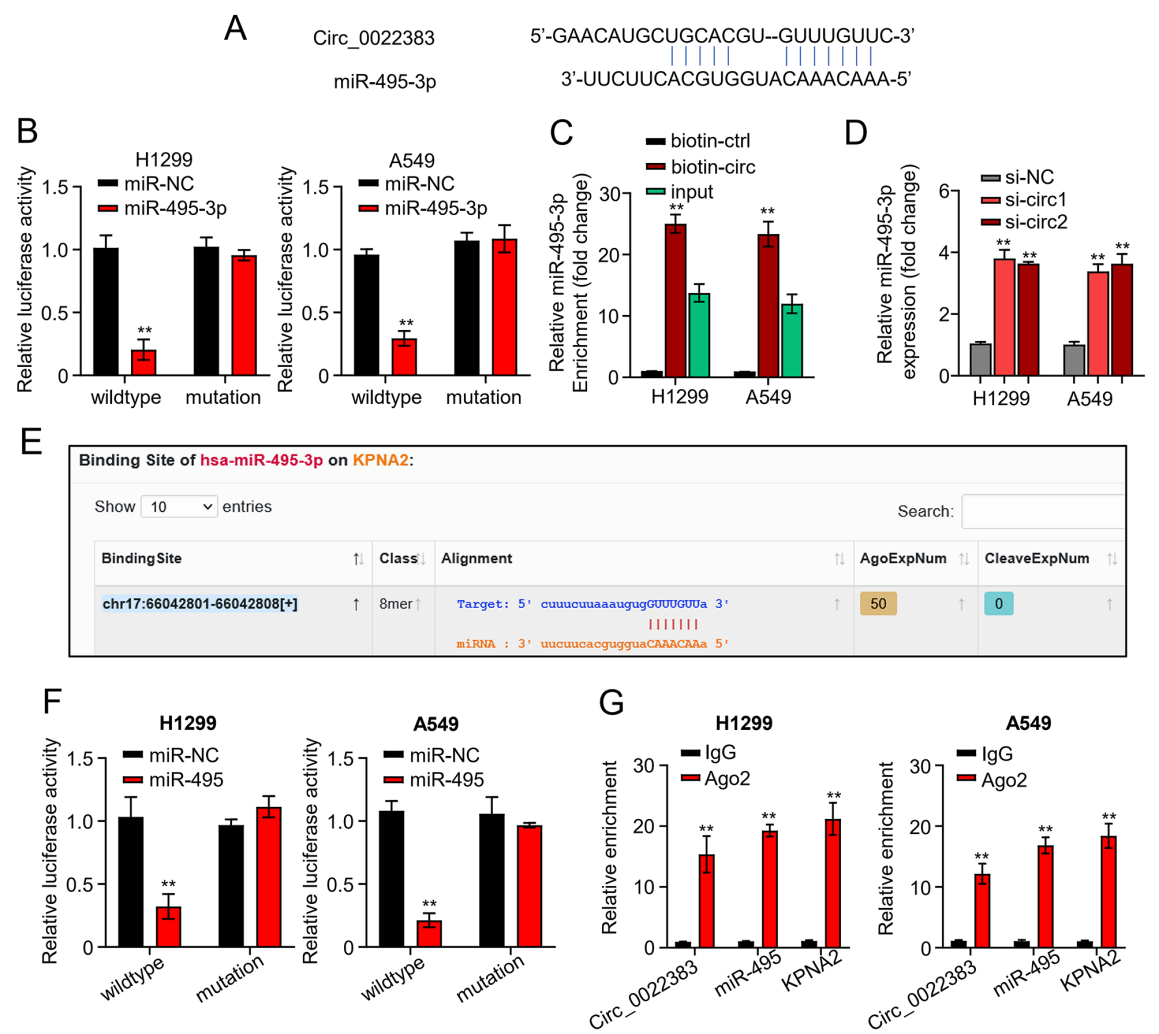
Hsa_circ_0022383 regulates KPNA2 expression by sponging miR-495-3p in NSCLC cells. **A**. MiR-495-3p was predicted as a potential target of circ_0022383. **B**. Relationship between circ_0022383 and miR-495-3p was examined by the luciferase reporter assay. **C**. Relationship between circ_0022383 and miR-495-3p were investigated through the RNA pull-down assay. **D**. MiR-495-3p expression in NSCLC cell lines (H1299 and A549) transfected with si-NC or si-circ_0022383 was calculated by qRT-PCR. **E**. The complementary sequences of miR-495-3p and KPNA2 predicted by the StarBase database. **F**. Relationship between miR-495-3p and KPNA2 was calculated by the Luciferase reporter assay. **G**. Relationship among circ_0022383, miR-495-3p and KPNA2 was calculated by the RNA immunoprecipitation (RIP) assay. All experiments were carried out in triplicate. “**” represents *P* < 0.01

We used the circular interactome database (https://circ-interactome.nia-nih.gov/) to screen the potential target of circ_0022383. We found that miR-495-3p was a putative miRNA targeted by circ_0022383 (Fig. 4A). To confirm the relationship between circ_0022383 and miR-495-3p, we performed the luciferase reporter and RNA pull-down assays. The luciferase reporter assay displayed that co-transfection of WT-circ_0022383 with miR-495-3p mimics to A549 and H1299 cells diminished the luciferase reporter activity, but co-transfection with MUT-circ_0022383 with miR-495-3p mimics did not (Fig. 4B). The RNA pull-down assay revealed that miR-495-3p was detected together with the circ_0022383-biotinylated probe but not with the NC probe (Fig. 4C). Moreover, we detected the expression level of miR-495-3p in A549 and H1299 cells transfected with si-NC or si-circ_0022383 using qRT-PCR. Silencing of circ_0022383 increased miR-495-3p expression levels in both cell lines (Fig. 4D). Furthermore, we screened the StarBase database and found that *KPNA2* was a potential gene targeted by miR-495-3p (Fig. 4E). The luciferase reporter assay showed that co-transfection of WT-KPNA2 with mimics control or miR-495-3p mimics reduced the luciferase reporter activity in A549 and H1299 cells, but co-transfection of MUT- KPNA2 did not (Fig. 4F). Additionally, the RIP assay revealed that Ago2 antibody precipitated Ago2 protein from the cell lysates and that circ_0022383, miR-495-3p, and KPNA2 were enriched in the Ago2 pellet (Fig. 4G). Collectively, our results demonstrate that circ_0022383 sponges miR-495-3p, thereby regulating KPNA2 expression in NSCLC cells.

### Circ_0022383 promotes NSCLC progression via miR-495-3p /KPNA2 signaling

**Fig. 5:**
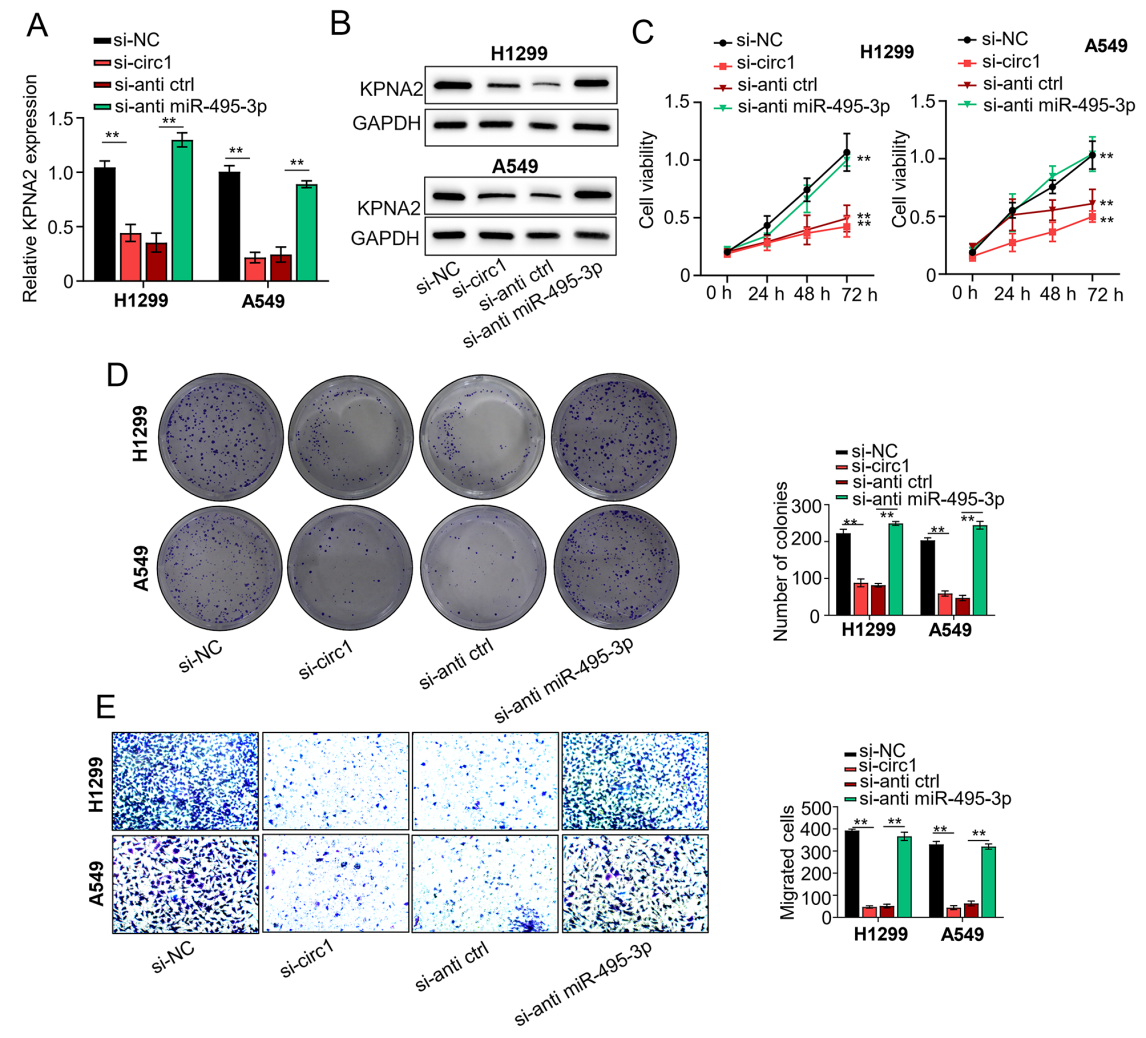
Hsa_circ_0022383 promotes NSCLC progression via miR-495-3p /KPNA2 signaling **A**. *KPNA2* mRNA expression in NSCLC cell lines (H1299 and A549) transfected with si-NC, si-circ_0022383, si-circ_0022383 + inhibitor control, or si-circ_0022383 + miR-495-3p inhibitor was determined by qRT-PCR. **B**. KPNA2 protein levels in (A) were determined by Western blot. **C**. Cell proliferation in (A) was determined by the CCK-8 assay. **D**. Cell colony number in (A) was calculated by the colony formation assay. **E**. Cell migration capability in (A) was determined by the Transwell assay All experiments were carried out in triplicate. “**” represents *P* < 0.01

**Fig. 6:**
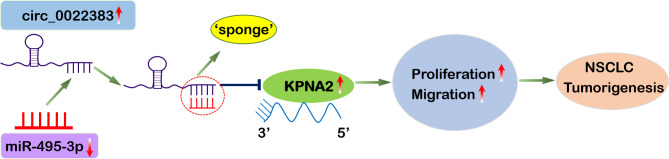
Schematic diagram of the function of circ_0022383/miR-495-3p/KPNA2 axis in NSCLC progression

To investigate whether circ_0022383 regulates NSCLC progression through modulating the miR-495-3p /KPNA2 signaling, we silenced circ_0022383 by transfecting A549 and H1299 cells with si-NC or si-circ_0022383. Silencing of circ_0022383 significantly increased the mRNA and protein levels of KPNA2, but this effect was abrogated by co-transfection with miR-495-3p inhibitor (Fig. 5B). In the mouse model, silencing of circ_0022383 repressed cell colony formation, proliferation, and migration abilities; however, this inhibiting effect was reversed by co-transfection with miR-495-3p inhibitor (Fig. 5C-E). These data suggested that circ_0022383 regulated the proliferation, migration, and invasion of NSCLC cells by modulating the miR-495-3p/KPNA2 axis (Fig. 6).

## Discussion

The aberrant expression of ncRNAs is closely associated the tumorigenesis and progression of malignant tumors [33]. Recently, circRNA has emerged as a new research hotspot in ncRNA biology. CircRNAs were previously deemed as the products of aberrant splicing events with limited functions [34, 35]. With the development of high-throughput RNA-sequencing and specific bioinformatic analysis, circRNAs have been reported as potent gene expression regulators in eukaryote cells, and many circRNAs have been implicated in tumor progression [36]. Due to their unique molecular structure and cell/tissue-specific expression, circRNAs are potential therapeutic drug targets and early diagnosis biomarkers for malignant tumors [8, 37]. However, the expression and function of most circRNAs during NSCLC tumorigenesis are not fully understood.

Here, we explored circRNA expression profiles in primary NSCLC tissues and adjacent nontumorous tissues from 5 patients by screening the GEO database. We homed in on circ_0022383 which was highly expressed in NSCLC tissues and predicted poor prognosis. Further, our results revealed that silencing of circ_0022383 repressed cell proliferation and metastasis in vitro and inhibited tumor metastasis and oncogenesis in vivo. In addition, we demonstrate that circ_0022383 sponges miR-495-3p to modulate the expression of KPNA2, contributing to NSCLC tumorigenesis and progression.

CircRNAs act as ceRNAs to sponge miRNAs to modulate gene expression [38, 39]. CircRNAs have been involved in the tumorigenesis of diverse malignant tumors, such as NSCLC. For instance, circUSP7 induces anti-PD1 resistance and CD8 + T cell dysfunction by targeting miR-934 in NSCLC [40]. CircRNA_103993 facilitates cell proliferation and apoptosis via sponging miR-1271 in NSCLC [41]. CircRNA_100565 promotes the cisplatin resistance of NSCLC cells by regulating cell proliferation and autophagy via targeting miR-337-3p [42]. Here we screened the circular interactome database and found that miR-495-3p was the potential target of circ_0022383. Through the luciferase reporter assay, RNA pull-down, and qRT-PCR, we confirmed that circ_0022383 directly targeted miR-495-3p and regulated its expression. Functionally, we confirmed that silencing of circ_0022383 repressed cell proliferation and metastasis, which was reversed by co-transfection with miR-495-3p inhibitor. Together, we demonstrate that circ_0022383 regulates NSCLC tumorigenesis through sponging miR-495-3p.

Typically, miRNAs act as potent gene expression regulators [43]. In this study, taking advantage of the StarBase database, the Luciferase reporter assay, and the RIP assay, we confirmed that *KPNA2* was the target gene of miR-495-3p. Consistent with the role of circRNAs as competing endogenous RNAs, our results also confirmed that circ_0022383 modulated KPNA2 expression by targeting miR-495-3p.

## Conclusion

In conclusion, we find that a novel circRNA, circ_0022383 was highly expressed in NSCLC tissues, and its expression is closely associated with the poor prognosis in patients. Moreover, we demonstrate that circ_0022383 functions as a ceRNA to sponge miR-495-3p to modulate KPNA2 expression in NSCLC. Our study sheds new light on the molecular mechanism of NSCLC tumorigenesis, providing a potential target for the diagnosing and treating NSCLC.

### Electronic supplementary material

Below is the link to the electronic supplementary material.

Supplementary Material 1

## Data Availability

The datasets used and/or analyzed during the current study are available from the corresponding author on reasonable request.
