# Supplementary material for: Hsa_circ_0022383 promote non-small cell lung cancer tumorigenesis through regulating the miR-495-3p/KPNA2 axis
Source: Cancer Cell Int. 2023 Nov 19;23:282. doi: 10.1186/s12935-023-03068-5 (PMC10658815; doi:10.1186/s12935-023-03068-5)
Supplement: Supplementary file 1 — Supplementary Material 1 [file 12935_2023_3068_MOESM1_ESM.pptx]

## Slide 1
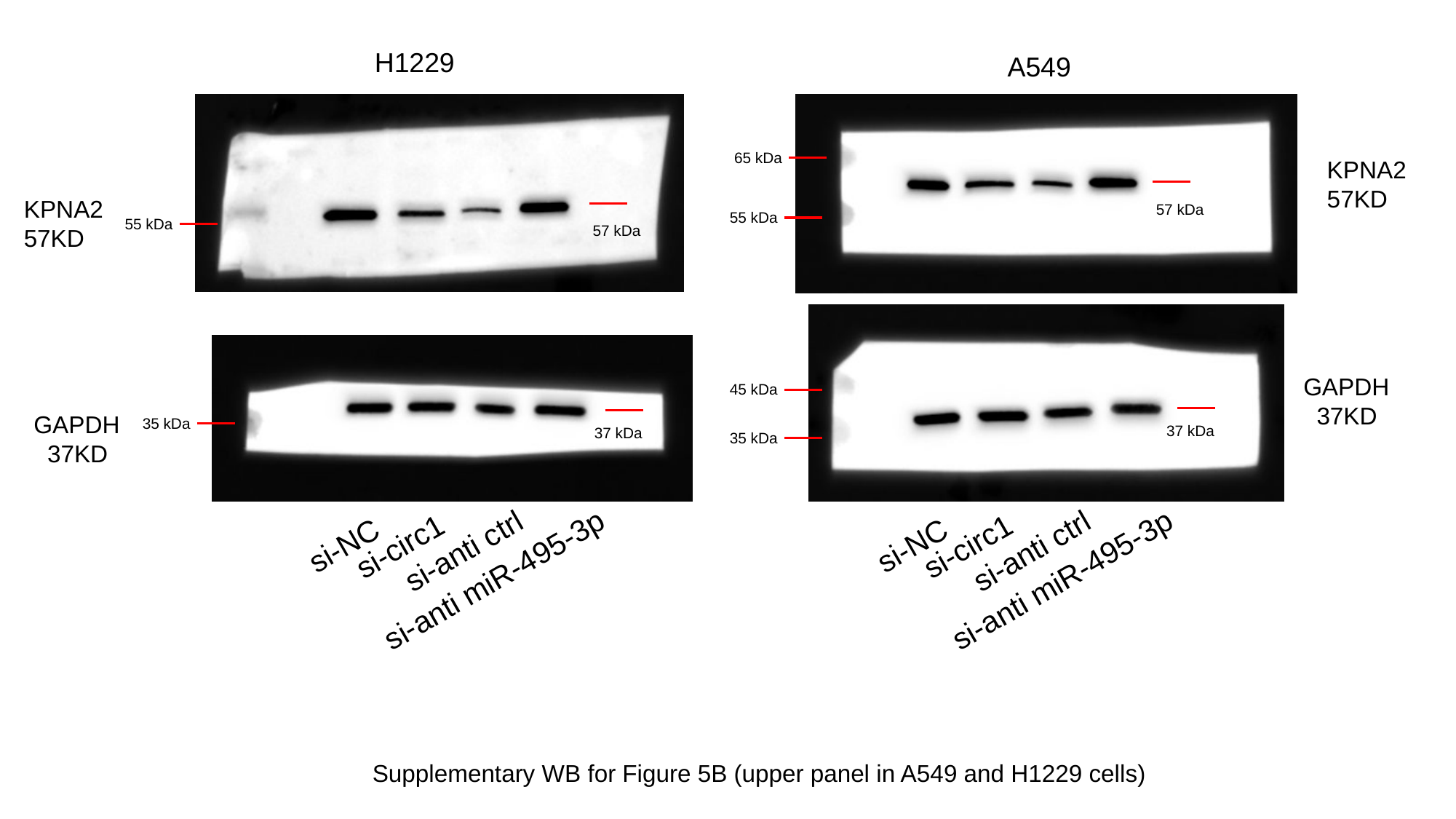

H1229
A549
65 kDa
KPNA2
57KD
KPNA2
57KD
57 kDa
55 kDa
55 kDa
57 kDa
GAPDH
 37KD
45 kDa
GAPDH
 37KD
35 kDa
37 kDa
37 kDa
35 kDa
si-NC
si-NC
si-circ1
si-circ1
si-anti ctrl
si-anti ctrl
si-anti miR-495-3p
si-anti miR-495-3p
Supplementary WB for Figure 5B (upper panel in A549 and H1229 cells)
